# Integration of AI-Based Synthetic CT Generation and Auto-Segmentation for CBCT-Guided Adaptive Radiotherapy in Prostate Cancer: A Feasibility Study

**DOI:** 10.3390/cancers18162607

**Published:** 2026-08-13

**Authors:** Xin Feng, Wenwen Zhang, Fukui Huan, Yuxiang Liu, Deqi Chen, Huan Chen, Ningyu Wang, Qian Liu, Huijuan Peng, Guodong Jin, Jianrong Dai, Yueping Liu, Kuo Men

**Affiliations:** National Cancer Center/National Clinical Research Center for Cancer/Cancer Hospital, Chinese Academy of Medical Sciences and Peking Union Medical College, Beijing 100021, China; fengxin930403@163.com (X.F.); zhangww_cicams@163.com (W.Z.); huanfukui@163.com (F.H.); yuxliu2021@outlook.com (Y.L.); michaelchan9412@outlook.com (D.C.); cxhkyk@163.com (H.C.); wny820588019@163.com (N.W.); liuqian202506@163.com (Q.L.); phj010203@163.com (H.P.); jgdwhued@163.com (G.J.); dai_jianrong@cicams.ac.cn (J.D.)

**Keywords:** artificial intelligence, synthetic CT, auto-segmentation, prostate cancer, adaptive radiotherapy

## Abstract

**Background/Objectives**: Adaptive radiotherapy (ART) is essential yet expensive in prostate cancer treatment. This study aims to preliminarily validate the feasibility of an integrated AI workflow for CBCT-guided adaptive radiotherapy in prostate cancer, and to provide a technical foundation for subsequent clinical translation. **Methods**: Planning CT and CBCT images from 120 patients were used for training and validation, while 21 patients were reserved for testing. A CycleGAN-ResNet generated synthetic CT (sCT) from CBCT, and an nnU-Net model performed auto-segmentation on the sCT. Image quality and segmentation accuracy were quantitatively assessed. The original plan was recalculated on sCT to evaluate actual dose delivery; if clinical constraints were unmet, adaptive re-optimization was performed, and plans were compared. **Results**: The total time per fraction in this study was approximately 19 ± 6 min, falling within the reported feasibility range for online ART. sCT image quality was significantly improved, making them suitable for subsequent auto-segmentation and treatment planning. The auto-segmentation technique substantially enhanced contouring efficiency, with the automatically generated contours requiring only minor modifications to meet clinical standards. In dosimetric analysis, the adaptive plans provided superior target coverage, CI, and HI. Compared with the actual dose delivered by the original plan, the adaptive plans yielded lower bladder V40 and lower rectal mean dose/V30/V40/V50. **Conclusions**: This study preliminarily validated the feasibility of an integrated AI workflow that concatenates CycleGAN-based sCT generation, nnU-Net-based auto-segmentation, and sCT-based adaptive plan re-optimization. Compared with conventional segmented studies, this integrated exploration facilitates a more comprehensive assessment of the potential value of AI technologies in CBCT-guided prostate cancer ART, offering a preliminary solution for promoting a cost-effective adaptive radiotherapy approach.

## Simple Summary

Adaptive radiotherapy is important for prostate cancer treatment because it adapts the plan to daily anatomical changes, but the equipment currently used for adaptive radiotherapy is very expensive, limiting its use in many hospitals. We developed an artificial intelligence-driven workflow that works with standard cone-beam CT (CBCT) images from conventional linear accelerators. This workflow converts CBCT into high-quality sCT for auto-segmentation and dose calculation, automatically delineates the target volumes and organs at risk, and re-optimizes the treatment plan when the initial plan no longer meets clinical requirements. This integrated approach is feasible and provides a cost-effective solution that could make adaptive radiotherapy more widely available using standard equipment.

## 1. Introduction

Prostate cancer is the most common malignant tumor of the male genitourinary system. According to GLOBOCAN 2022 statistics from the World Health Organization, its incidence ranks second and its mortality ranks fifth among males globally [1]. Radiotherapy is a cornerstone of prostate cancer treatment. Precise dose delivery during radiotherapy is critical for effective disease control; however, irregular prostate motion and daily variations in bladder and rectal filling complicate the achievement of such precision [2]. These inter-fractional uncertainties can significantly affect dosimetric outcomes. To mitigate the risk of target underdosage, expanded planning target volume (PTV) margins are commonly employed, but this often increases the dose to organs at risk (OARs), particularly the bladder and rectum, leading to gastrointestinal and genitourinary toxicities [3].

Adaptive radiotherapy (ART) leverages advanced imaging technologies to modify the treatment plan based on anatomical and even biological changes observed during the treatment course, aiming to precisely irradiate the target while enhancing OAR sparing [4,5]. However, implementing ART often requires dedicated and expensive equipment, and the additional imaging examinations involved may increase the financial burden on patients or deliver unnecessary radiation dose [6,7]. If ART could be performed using the onboard CBCT system, this problem could be largely resolved, enhancing the generalizability of the technique. Currently, however, most CBCT images still have certain limitations that restrict their application in ART [8]. The primary challenges are poor image quality and inaccurate electron density information [9]. Poor image quality makes accurate target identification difficult, while inaccurate electron density precludes precise dose calculation, prompting extensive efforts to improve image quality and Hounsfield unit (HU) accuracy [10,11]. A large body of research now indicates that deep-learning-processed CBCT images can meet clinical requirements [12,13]. Another major obstacle to the widespread adoption of ART is the cumbersome clinical contouring process. Manual contouring can take radiation oncologists tens of minutes or even hours, substantially reducing the feasibility of implementing ART. Furthermore, manual contouring is subject to inter-observer variability influenced by expertise and experience, and these inconsistencies can affect subsequent dosimetric analyses [14]. In recent years, artificial intelligence has played an increasingly vital role in medical image analysis and clinical decision-making [15,16,17,18,19]. AI-driven automated and personalized delineation methods have significantly improved efficiency, thereby accelerating the clinical deployment of ART [20]. With a variety of algorithms emerging, the next frontier lies in integrating these artificial intelligence (AI) modules into a seamless clinical workflow—a step critical for translating technological advances into tangible patient benefits.

Currently, most studies focus on a single component of the workflow. Research that integrates sCT generation, auto-segmentation, and adaptive re-planning within a unified framework for holistic validation remains scarce. The core innovation of this study lies in exploration at the integration level: first, concatenating the three modules into an end-to-end workflow for overall validation; second, relying entirely on CBCT images from conventional C-arm linear accelerators, using AI to compensate for image quality deficiencies without hardware upgrades, thereby significantly lowering the barrier to ART implementation. This study aims to preliminarily validate the feasibility of this integrated AI workflow in CBCT-guided ART for prostate cancer and to provide a technical foundation for subsequent clinical translation.

## 2. Materials and Methods

### 2.1. Patient Data

This study included 141 prostate cancer patients who received radiotherapy at our institution. Images from 120 patients were used for training and validation, while images from 21 patients were used exclusively for testing. Of these, 120 patients were randomly assigned to model development, which was further divided into a fixed training set (90 patients) and a fixed internal validation set (30 patients). The validation set was used for hyperparameter tuning and early stopping during training. The remaining 21 patients were completely held out as an independent test set and were not involved in any training or validation steps. To prevent patient-level data leakage, all daily CBCT fractions from the same patient were strictly kept within the same set (training, validation, or test). The model’s final performance was evaluated on the independent test set only after all development and tuning were completed. All participants underwent CBCT imaging before each treatment fraction. The study protocol was reviewed and approved by the Institutional Ethics Committee. All patients underwent contrast-enhanced CT simulation for treatment planning using a Brilliance Big Bore CT simulator (Philips Medical Systems, Cleveland, OH, USA) under physician supervision. Before imaging, patients were instructed to reduce bowel gas through dietary modification, empty the rectum, and maintain a comfortably full bladder. The contrast-enhanced CT scan slice thickness was 3 mm, with the scan range extending from the L4 vertebra to 3 cm below the ischial tuberosities.

The acquired images were imported into the Pinnacle treatment planning system for contouring. Regions of interest (ROIs) were manually delineated by a radiation oncologist following the ESTRO ACROP consensus guideline for target volume delineation and the RTOG consensus guideline for OAR contouring [21,22], and subsequently reviewed and confirmed by a senior radiation oncologist. The clinical target volume (CTV) consisted of the prostate and seminal vesicles depending on disease stage. The PTV was generated by adding a 5 mm margin in the left-right and anterior directions and a 3 mm margin in the craniocaudal and posterior directions to the CTV. For patients with lymph node involvement, the pelvic drainage region was additionally contoured as CTV1, and the corresponding PTV1 was generated by adding a 5 mm isotropic margin. Treatment plans were delivered on a Varian Edge linear accelerator (Varian Medical Systems, Palo Alto, CA, USA) using the volumetric modulated arc therapy (VMAT) technique in flattening-filter-free (FFF) mode.

### 2.2. Image Processing

#### 2.2.1. CBCT Acquisition

Before each treatment, patients followed the same bladder and bowel preparation protocol as during CT simulation. Once readiness was confirmed, patients were positioned to reproduce the simulation setup. Daily online CBCT scans were acquired on the Varian EDGE linear accelerator using a pelvic imaging protocol with exposure settings of 125 kV and 1080 mAs. The acquired CBCT images were then rigidly registered to the planning CT images using a six-degree-of-freedom registration.

#### 2.2.2. sCT Generation

**Figure 1:**
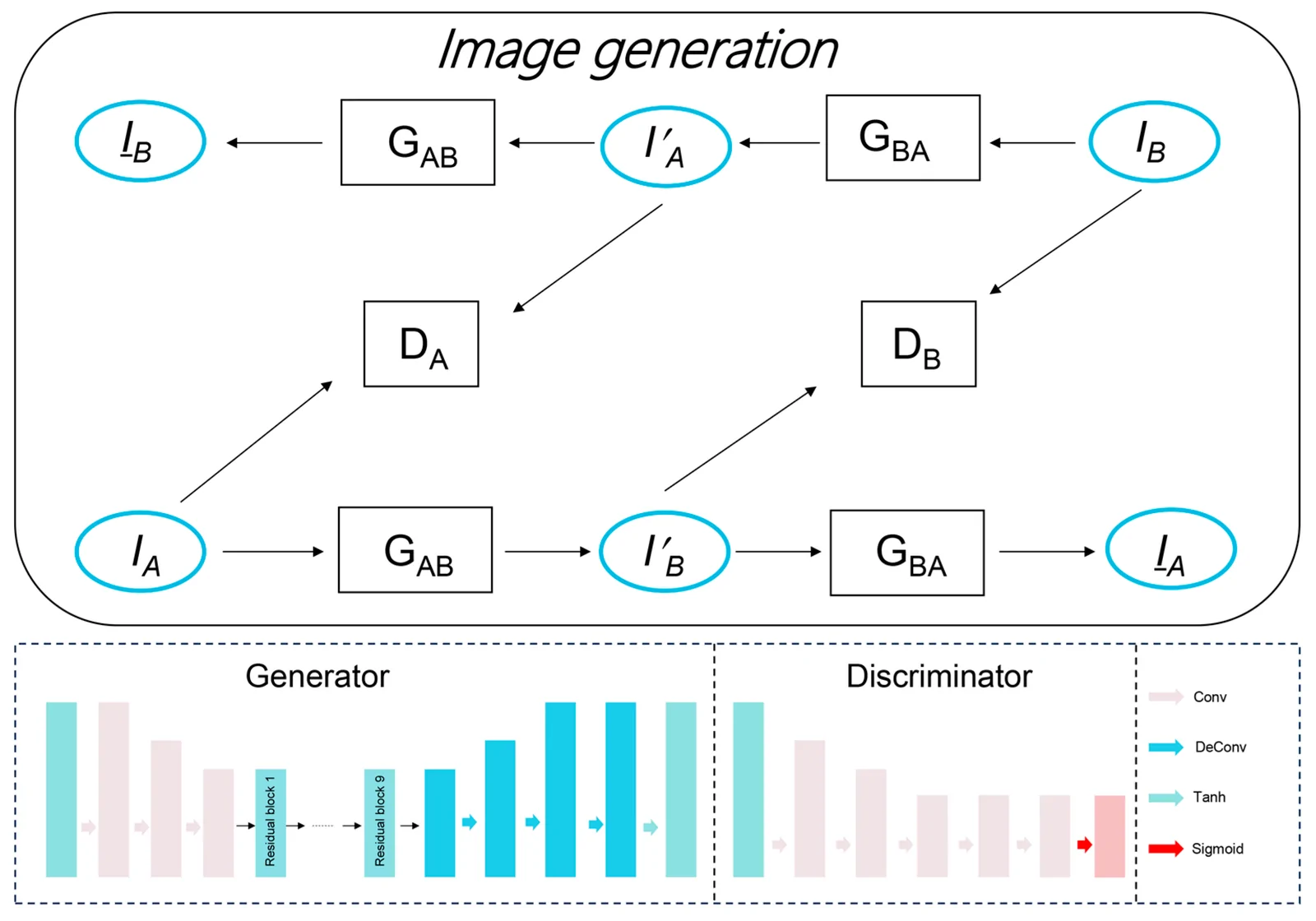
Schematic diagram of the CycleGAN architecture for image generation. The figure contains a forward loop and a reverse loop, with the forward loop direction from IA to IA and the reverse loop direction from IB to IB. In the training process, generator GAB transforms images from domain A into synthetic domain B (I’B), while discriminator DB distinguishes between real and synthetic B, forming the adversarial loss. The synthetic B output is then passed through generator GBA to reconstruct domain A (IA), enabling calculation of the cycle-consistency loss between reconstructed A and real A, thereby preserving domain A’s structural integrity. The CycleGAN framework incorporates two such cycles to learn bidirectional mappings between the two domains.

The patient’s pCT and daily CBCT images were imported into MIM software (version 6.7, MIM Software Inc., Cleveland, OH, USA) and aligned through rigid registration. Registration was performed by a senior radiation therapist and subsequently reviewed by a physician. Images were preprocessed using min-max normalization to the [−1, 1] range before being fed into the network. A CycleGAN framework, an unsupervised image-to-image translation generative adversarial network that enables cross-domain conversion without paired training data [23], was used to generate sCT images from daily CBCT. Specifically, a CycleGAN-ResNet architecture (see Figure 1 for details) was adopted for unsupervised image translation, incorporating a ResNet generator with nine residual blocks and a PatchGAN discriminator. The model was trained on an NVIDIA V100 GPU and subsequently used for inference. After training, inference for each case was completed within seconds. Since no ground truth image exists for image synthesis, a deformed CT image generated using a B-spline deformable image registration algorithm served as the reference standard for evaluating sCT image quality [24]. We acknowledge that this reference is not an independent ground truth, as it may contain registration errors and may not fully capture bowel gas changes or organ deformations. However, acquiring daily verification CT for every fraction is clinically impractical due to additional radiation exposure. Thus, this approach remains widely adopted in the literature, and our primary endpoint was dose calculation accuracy, which we directly assessed via gamma analysis, a clinically established metric for ART dose validation. Future validation using phantom-based studies or repeated CT on selected fractions could further strengthen the evidence. Detailed network architecture parameters are provided in Appendix A.1.

### 2.3. Auto-Segmentation

Using the CycleGAN-generated sCT images as input, the nnU-Net model was employed for multi-organ segmentation [25]. This deep-learning-based segmentation method can automatically configure itself—including preprocessing, network architecture, training, and postprocessing—for any new task and has been widely used in medical image segmentation, outperforming many state-of-the-art networks [26]. After the population model was trained, lightweight fine-tuning was performed using each patient’s pCT and manual contours as individual priors, after which the model was applied to segment sCT images of subsequent fractions. This fine-tuning process was performed only once before the patient’s first treatment session. Before each subsequent fraction, the generated sCT image was directly fed into this fine-tuned personalized model to rapidly and accurately output ROIs adapted to the individual patient. Detailed network architecture parameters are provided in Appendix A.2.

The segmentation model delineated ROIs on sCT images, including the CTV, bladder, rectum, colon, small intestine, and femoral heads. All contours were subsequently reviewed and adjusted by experienced physicians and finally verified by a radiation oncologist to minimize inter-observer variability. The specific manual review and editing procedure was as follows: the physician opened the sCT image and the automatically generated contours in dedicated radiotherapy software. The physician first reviewed all contours globally to check for obvious misplacements or omissions. Then, for each ROI, the alignment between the contour boundary and the anatomical structure on the image was checked slice by slice, with local adjustments made for regions where the deviation exceeded 1 mm. After modification, a senior physician conducted a secondary review. The time spent on the entire review and editing process was recorded for subsequent efficiency analysis.

### 2.4. Adaptive Planning

**Figure 2:**
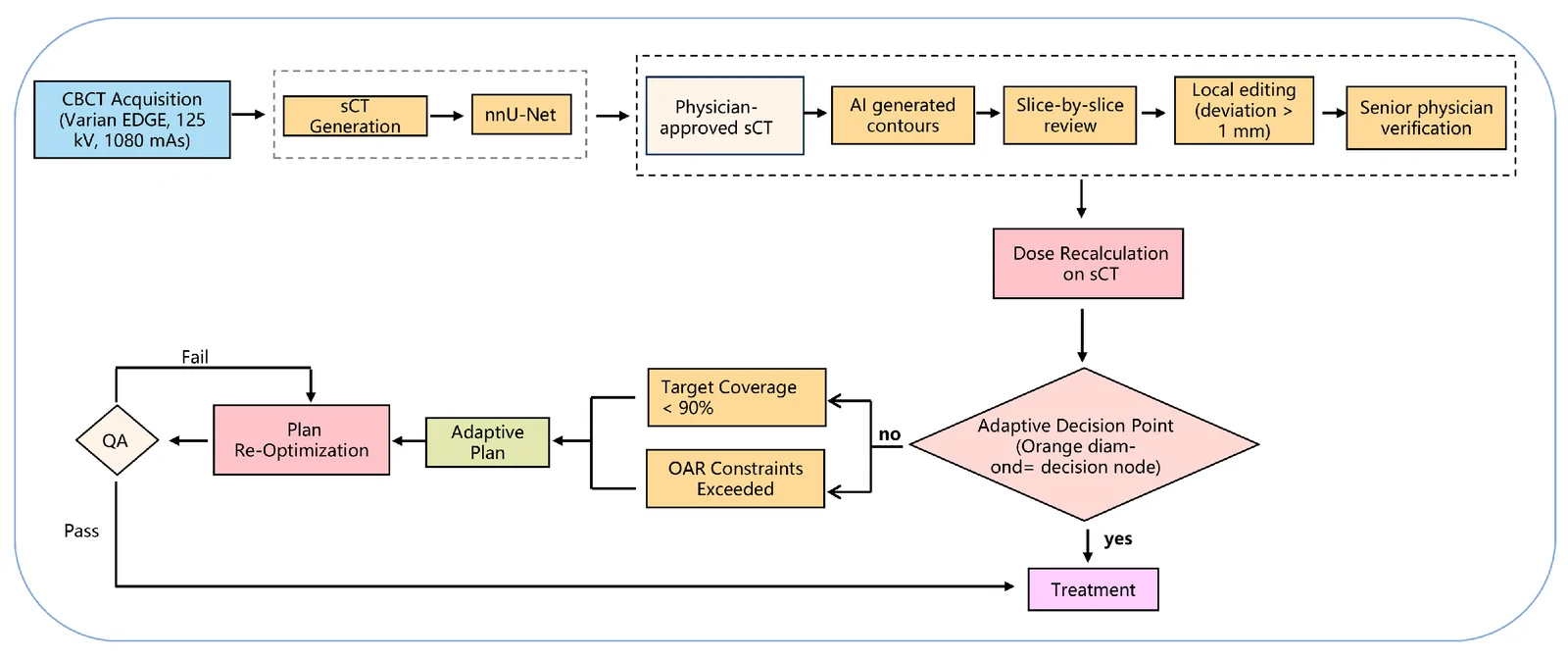
The process begins with CBCT image acquisition, followed by sCT generation using the CycleGAN method. Automatic delineation is then performed with AI-based tools, and the contours are modified and approved by physicians before use in plan evaluation. The original treatment plan is transferred to the sCT, with setup error correction applied for clinical assessment. If the recalculated dose distribution meets clinical requirements, treatment proceeds directly. If not, adaptive planning and quality assurance (QA) are conducted.

Given that this is a feasibility analysis aimed at preliminary validation of the technical feasibility and clinical workflow operability of the integrated workflow, rather than evaluating cumulative dose over the full treatment course, adaptive plan design and dosimetric comparison were based on a single fraction image per patient. The specific procedure was as follows: for each patient, the contoured sCT image from a randomly selected fraction was imported into the Pinnacle system. The initial plan was generated by recalculating the dose on the sCT image using the prescription parameters of the original plan based on the pCT, after correcting for setup errors through image registration. The purpose of this step was to evaluate the actual dose distribution the patient would receive under the current anatomical state if treated according to the original plan. Assessing the dosimetric metrics of this initial plan allowed determination of whether the original plan still met clinical requirements in the current anatomy. This “virtual dose verification” is a key basis for adaptive radiotherapy decision-making. If all metrics of the initial plan satisfied clinical requirements, the original plan was maintained; if the target coverage fell below 90% or any OAR dose constraint was exceeded by more than 10% of the prescribed limit, adaptive plan re-optimization was initiated. The re-optimized plan had to pass independent three-dimensional dose verification based on a Monte Carlo algorithm before it could be used for treatment. If verification failed, the plan would be modified and re-verified before clinical use. The adaptive re-planning trigger thresholds (target coverage < 90% or OAR constraints exceeded) were derived from our institutional clinical protocol, guided by ICRU Report 83, QUANTEC, and RTOG consensus guidelines. We note that specific thresholds may vary across institutions, and our upcoming multi-centre trial will evaluate the impact of alternative thresholds on clinical outcomes. The detailed workflow is shown in Figure 2.

### 2.5. Evaluation

sCT image quality was compared against the deformed CT images using peak signal-to-noise ratio (PSNR), mean absolute error (MAE), structural similarity index (SSIM), and gamma pass rate. The global 3D gamma pass rate was calculated using Python 3.12.7 with a low-dose threshold set at 10%. The accuracy of auto-segmentation was evaluated by comparing the automatic contours with the manually refined contours using the Dice similarity coefficient (DSC), 95% Hausdorff distance (HD95), and mean surface distance (MSD). The detailed calculation methods for these metrics are provided in Appendix B.

Differences between the Adaptive Plan and Initial Plan were analyzed using dose volume histogram (DVH), consistency index (CI), and homogeneity index (HI). HI was calculated, following Wu et al.’s method [27]:

(1) $$\mathrm{HI}=\frac{D_{2\%}-D_{98\%}}{D_{p}}\;$$

where D2% and D98% are the doses to 2% and 98% of the target volume, and Dp is the prescribed dose.

CI was calculated by following the method of van’t Riet et al. [28]:

(2) $$CI=\frac{V_{T,\mathrm{ref}}}{V_{T}}\times \frac{V_{T,\mathrm{ref}}}{V_{\mathrm{ref}}}\;$$

where VT, ref is the volume of the target receiving a dose equal to or greater than the reference dose, VT is the target volume, and Vref is the treated volume of the reference isodose.

### 2.6. Statistical Analysis

Continuous variables were expressed as mean ± standard deviation or median (interquartile range) depending on data normality. Data analysis was performed using SPSS version 22.0 (IBM Corp., Armonk, NY, USA). For normally distributed data, the paired *t*-test was applied; for non-normally distributed data, the Wilcoxon signed-rank test for paired samples was used. A *p*-value < 0.05 was considered statistically significant.

## 3. Results

**Figure 3:**
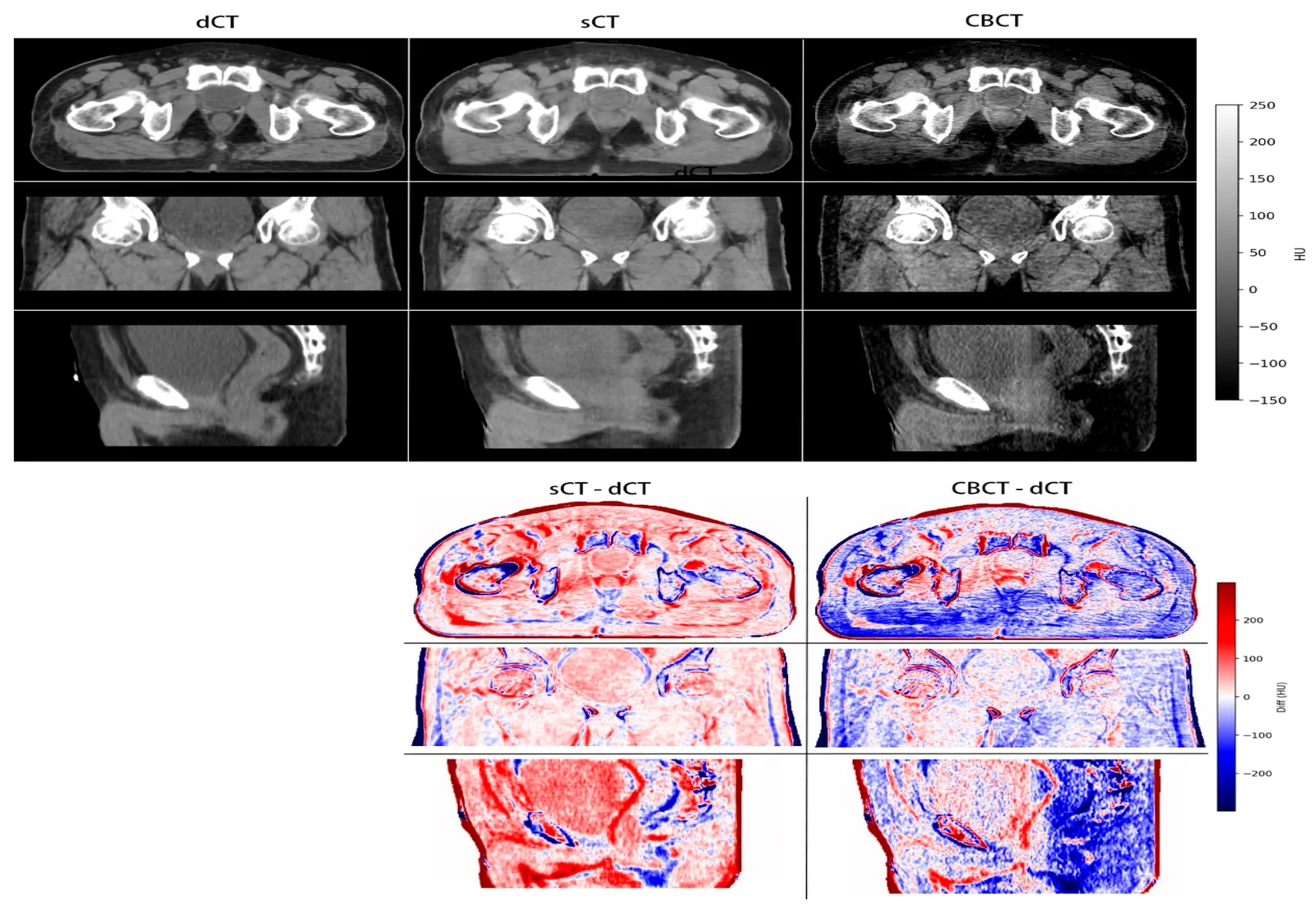
CBCT, sCT, and deformed CT images and HU difference in a prostate cancer patient. Compared with CBCT, the sCT images show markedly improved quality, with reduced noise and artifacts, and demonstrate closer consistency with the deformed CT reference standard.

The trained model generated sCT images within seconds. The sCT images preserved the anatomical fidelity of the original CBCT while reducing artifacts, thereby improving image quality (Figure 3). sCT markedly enhanced image sharpness and HU accuracy. To quantify the improvement over raw CBCT, we calculated the same image-quality metrics between the original CBCT and the deformed CT reference. The results demonstrated that sCT significantly outperformed raw CBCT across all metrics: MAE decreased from 32.52 ± 3.75 HU to 28.85 ± 3.68 HU, PSNR increased from 22.00 ± 1.21 dB to 23.14 ± 1.80 dB, and SSIM increased from 0.79 ± 0.02 to 0.84 ± 0.02. For dose calculation verification, the gamma pass rates were 96.86% ± 1.96% under the 1%/1 mm criterion and 98.51% ± 1.26% under the 2%/2 mm criterion. These findings indicate that the sCT images meet clinical requirements and are suitable for ROI delineation and treatment planning.

**Figure 4:**
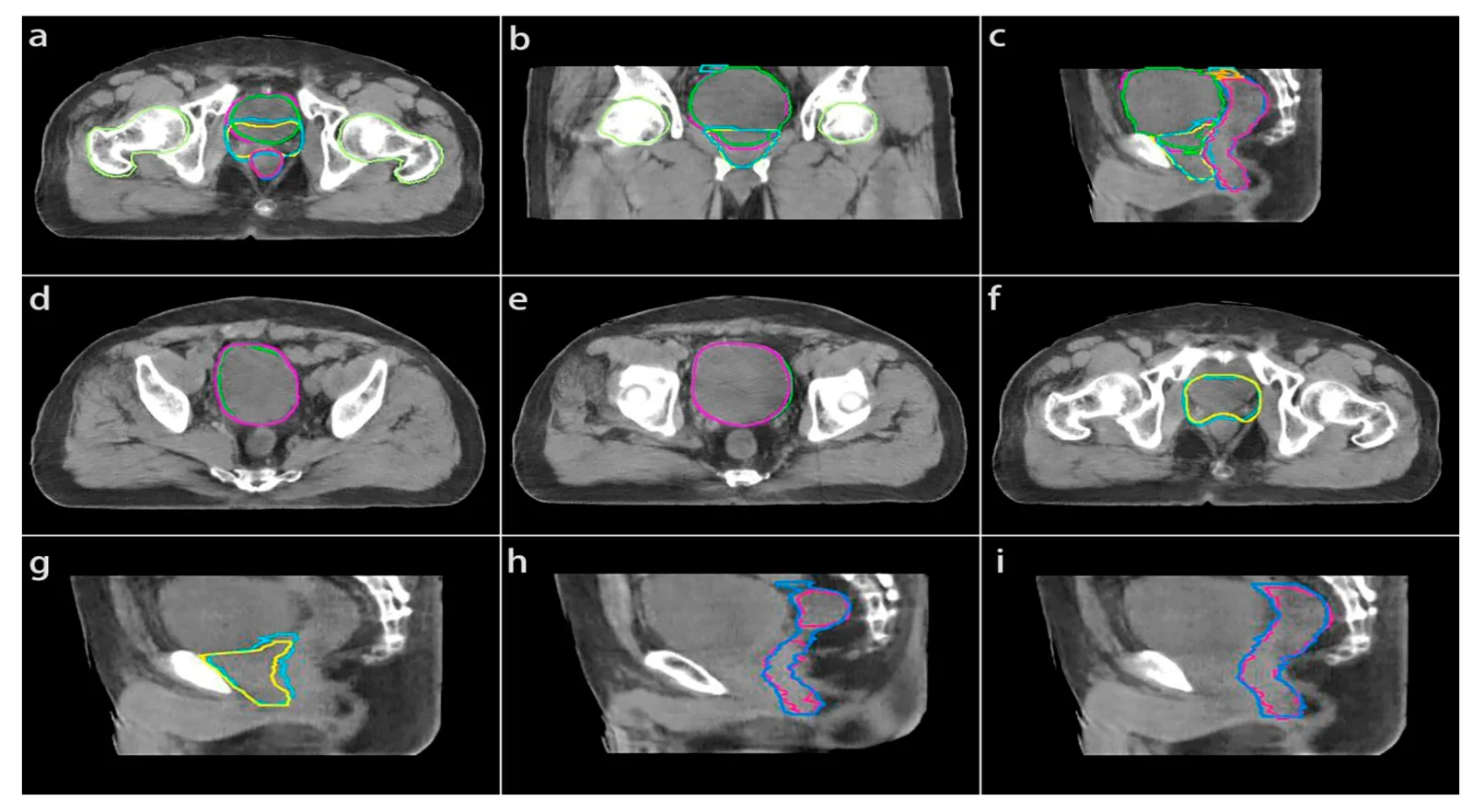
Example of auto-segmentation on sCT images. Manually contoured structures are shown as follows: bladder (magenta), colon (cyan), CTV (yellow), femoral heads (red), and rectum (blue). Auto-segmented structures are represented as: bladder (green), colon (orange), CTV (light blue), femoral heads (lime), and rectum (deep pink). Panels (**a**–**c**) display comprehensive delineation results, while panels (**d**,**e**), (**f**,**g**), and (**h**,**i**) provide detailed comparisons of bladder, CTV, and rectum contours between manual and automatic delineation, respectively.

The proposed AI-based online ROI delineation method enabled fast and accurate segmentation. Figure 4 shows a comparison of the differences between automatic and manual segmentation. For the bladder and femoral heads, auto-segmentation performed excellently, requiring almost no manual correction. For the rectum and CTV, only minor adjustments were needed to meet clinical standards. This automated approach reduced the physicians’ workload and markedly improved overall workflow efficiency. On average, the AI-generated contours, after physician review, required 12.5 min per case, whereas completely manual segmentation required 69.5 min. A detailed comparison between automatic and manual delineation is presented in Table 1.

To assess robustness, we visually inspected all failure cases in the test set. The most common failure patterns included: (1) severe bowel gas causing segmentation errors in the intestine and colon; and (2) large anatomical deviations (e.g., extreme bladder filling) exceeding the training distribution, leading to reduced sCT quality. These cases accounted for approximately 5% of the test fractions and required more extensive physician correction.

Compared with the initial plan, the adaptive plan achieved superior target coverage, better normal tissue sparing, and higher overall plan quality, demonstrating the clinical value of re-optimization. CBCT-based prostate ART not only improved target coverage but also significantly reduced radiation exposure to critical organs such as the bladder and rectum. Among all the selected initial plans, no case achieved the 95% target coverage threshold. Moreover, several patients exceeded bladder constraints (5/21 patients V50 > 20%, 2/21 patients V40 > 30%, 6/21 patients V30 > 40%), and rectal constraints were also frequently violated (15/21 patients V50 > 10%, 13/21 patients V40 > 20%, 4/21 patients V30 > 40%), highlighting the limitations of the initial plans. Among the 21 test patients, adaptive re-optimisation was triggered in 14 patients (66.7%), primarily due to target coverage shortfalls (10/14, 71.4%), with the remaining 4 cases (28.6%) triggered by OAR constraint violations. The sCT-based adaptive plan showed statistically significant improvements (*p* < 0.05) across multiple endpoints, including target coverage, bladder V40, rectal V30, rectal V40, rectal V50, and rectal mean dose (Table 2). Although a few metrics showed slight decreases in individual fractions, these differences were not statistically significant and were within clinically acceptable limits.

## 4. Discussion

This study integratively validated an AI-driven ART workflow that is entirely based on CBCT from a conventional C-arm linear accelerator, encompassing the three core components of sCT generation, auto-segmentation, and adaptive re-optimization. We adopted a multi-metric evaluation framework to systematically validate this solution at three levels: image quality, contouring accuracy, and plan quality. The results demonstrate that this solution achieves clinical efficacy comparable to existing mainstream ART platforms with clear dosimetric benefits, while substantially lowering the technical barrier and economic cost, thereby laying the foundation for the broader implementation of prostate ART on standard C-arm linear accelerator platforms.

Currently clinically available ART primarily falls into two major technical pathways. One is the MR-linac platform, which enables precise online adaptation based on high-definition MR images but incurs extremely high equipment costs and lengthy treatment times. The other involves dedicated ART platforms based on CT or CBCT, such as the United Imaging uRT-linac 506c or the Varian Ethos system, but the installed base is limited, making large-scale deployment challenging. This solution is positioned as a third pathway: utilizing the onboard CBCT of conventional accelerators combined with AI enhancement to achieve online ART efficacy comparable to that of dedicated equipment without hardware upgrades, significantly improving accessibility and cost-effectiveness while maintaining a comparable time frame. It should be noted that dedicated platforms possess inherent advantages in system integration and cutting-edge development that this solution cannot match. Our goal is not to replace dedicated equipment but to provide a “sufficient and usable” alternative for the vast majority of conventional accelerators.

This study does not validate a single AI technology in isolation; rather, it concatenates sCT generation, auto-segmentation, and adaptive plan re-optimization within a unified framework for holistic validation. To the best of our knowledge, this is the first preliminary study integrating CycleGAN-based sCT generation with nnU-Net-based auto-segmentation for adaptive plan re-optimization in prostate cancer on a conventional linear accelerator platform. Compared with most studies focusing on a single component [29,30,31], this integrated exploration enables a more comprehensive assessment of feasibility and potential benefits on conventional C-arm linear accelerators, helping to accelerate clinical translation. It must be noted that this study did not create new generation or segmentation algorithms; rather, it adapted, concatenated, and performed end-to-end evaluation of existing state-of-the-art but often isolated tools (CycleGAN, nnU-Net). We argue that this exploration at the integration level carries equal importance to technical-level algorithmic innovation for promoting ART implementation in resource-limited centers. To better position our work within the current literature, we compared our integrated workflow with recently published ART frameworks, as summarized in Table 3.

At the technical level, we demonstrated that low-quality CBCT images can be converted into sCT images that meet dose calculation requirements using the CycleGAN model. In this study, the sCT images achieved an MAE of 28.85 ± 3.68 HU, a gamma pass rate of 96.86% ± 1.96% under the 1%/1 mm criterion, and 98.51% ± 1.26% under the 2%/2 mm criterion. These results are comparable to or better than those of previous studies [36,37]. All generated sCT images were reviewed by experienced radiation oncologists during contour verification, with no obvious anatomical hallucinations observed. We recognise that more rigorous validation, including deformable registration and landmark-based geometric checks, will be incorporated in future studies to further confirm anatomical consistency. All planning CT images used in this study were contrast-enhanced. Because the CycleGAN was trained using these images as the target CT domain, contrast enhancement may have affected the learned CT intensity distribution. Although the unpaired translation strategy and cycle-consistency constraint preserve the anatomy represented in the input CBCT and reduce the likelihood of introducing unrelated structures, they cannot completely exclude contrast-related intensity bias. Future studies should evaluate this effect by comparing models trained using contrast-enhanced and non-contrast CT datasets and by performing region-specific analysis in contrast-sensitive tissues. Meanwhile, the nnU-Net model generated clinically acceptable automatic contours for most ROIs. Although the opacity of deep learning models means that we cannot fully understand their internal working mechanisms, their outputs are efficient and practical. A senior radiation oncologist could easily verify and correct them, requiring only minor adjustments to meet clinical standards [38], which is more efficient than completely manual contouring. These results are consistent with prior studies [39]. It should be noted that in this study, using physician-corrected contours as the reference may introduce incorporation bias, which could affect the evaluation of the auto-segmentation algorithm. However, given our primary focus on workflow efficiency, we consider this approach acceptable for a feasibility study. In future validation, we will employ blinded independent manual contours as the reference. Recent advanced network architectures, including SwinUNet, TransUNet, MedNeXt, UNETR, and diffusion-based synthesis models, have exhibited superior performance in medical image analysis. Evaluating and integrating these cutting-edge models constitutes a key direction of our future work. Benefiting from the modular design of our current workflow, individual components can be seamlessly replaced with newly developed algorithms to further boost overall performance. Overall, AI technologies have unblocked two key bottlenecks in the technical chain of using CBCT for adaptive radiotherapy.

At the clinical evaluation level, by comparing the dosimetric parameters of the sCT-based adaptive plans with those of the initial plans, this study demonstrated that this integrated solution can generate improved adaptive plans on conventional CBCT platforms. Although daily image guidance can correct systematic setup errors, it cannot fully account for inter-fractional anatomical changes [40], and the comparison between sCT and pCT highlighted persistent inter-fractional differences. The dosimetric results of this study clearly validate the clinical necessity of ART in prostate cancer, and the observed benefits are comparable to the improvements reported previously [33,34,35]. The dosimetric improvements demonstrated in this study represent technical feasibility rather than clinical superiority. While improved target coverage and OAR sparing are widely accepted surrogate endpoints for predicting reduced toxicity and better tumour control, direct evidence of clinical benefit—such as reduced gastrointestinal/genitourinary toxicity, improved biochemical recurrence-free survival, or overall survival—requires prospective clinical trials with long-term follow-up. Future validation studies with clinical outcome measures are warranted before this workflow can be recommended for routine clinical deployment.

The overall workflow time is a core metric determining whether online ART can be integrated into the routine treatment schedule. The estimated total time in this study was approximately 19 ± 6 min, falling within the reported feasibility range for online ART, and it is expected to be further reduced with future implementation of background parallel computing. Specifically, CBCT acquisition and image registration required approximately 4 min. sCT generation via CycleGAN inference took approximately 0.5 min with ~8 GB GPU memory, and auto-segmentation via nnU-Net inference took approximately 1 min with ~3 GB GPU memory. Physician review and correction of the AI-generated contours required 12.5 min on average. Plan evaluation took approximately 2 min, and when adaptive re-optimisation was triggered, the optimisation process required approximately 10 min. The worst-case total time was 35 min, which occurred in patients requiring both extensive contour correction and plan re-optimisation. The overall time for the adaptive workflow was primarily spent on physician review and plan optimization. Because senior physicians are often busy, this study adopted a tiered review model of “slice-by-slice adjustment by a junior physician followed by final confirmation by a senior physician,” which to some extent prolonged the overall time. If the entire process had been independently completed by a senior physician, the total time could have been further compressed. Additionally, a learning effect analysis showed that the physician review time decreased from approximately 15 min in the early phase to under 10 min in the later phase, indicating that review efficiency improves continuously as physicians become familiar with the “style” of the automatic contours. Meanwhile, in terms of plan optimization, automated planning is also gradually evolving. With subsequent introduction of automatic plan design and evaluation metric models, enhancing system integration can further compress the time and promote the practical implementation of the technology.

Current research on adaptive radiotherapy for prostate cancer is predominantly concentrated on dedicated platforms [41,42,43]. The fact that this study does not rely on expensive or specialized equipment implies significant potential for dissemination to a wide range of medical institutions. This study provides multi-level quantitative evidence ranging from the pixel level (MAE/PSNR) and dose level (Gamma) to the plan level (DVH/CI/HI), relatively comprehensively demonstrating the clinical potential of this integrated solution.

This study is a retrospective study and is currently limited to offline data analysis. The overall workflow integration is relatively low, leaving room for time compression; further integration of automated modules is needed to shorten the time spent in each step. We acknowledge that the entire study was conducted using data from a single institution and a single linear accelerator platform. Deep learning models may suffer from performance degradation when applied to images from different scanners, reconstruction algorithms, or institutions. This limitation may impact the generalisability of our workflow in routine clinical deployment. To address this, we are currently preparing a prospective multi-centre study involving different linac vendors (Varian and Elekta) and imaging protocols to assess generalisability. Furthermore, domain adaptation techniques, such as fine-tuning on a small subset of target-domain data, will be implemented to mitigate performance degradation when deploying to new institutions. Furthermore, beyond technical performance, the translation of this workflow into routine clinical practice will require addressing practical challenges including regulatory approvals (e.g., FDA/CE clearance), seamless integration with existing commercial TPS and PACS, and departmental workflow restructuring. Nevertheless, the preliminary results of this study indicate that the integration of AI-based synthetic CT generation and auto-segmentation for CBCT-guided adaptive radiotherapy in prostate cancer is feasible.

In future work, we will validate and optimize this approach across multiple platforms and disease sites, introduce clearly defined decision thresholds and standardized response pathways, and enhance automation integration to reduce human intervention.

## 5. Conclusions

This proof-of-concept study demonstrates that combining AI-based sCT image generation with auto-segmentation can enable adaptive radiotherapy on C-arm linear accelerators, offering a potentially flexible and affordable alternative to dedicated adaptive platforms such as Ethos or MR-Linac. The improved soft-tissue contrast and CT number accuracy on sCT images allow direct dose calculation on CBCT images. This workflow enables timely adjustments of targets and OARs, improving target coverage while reducing dose to critical structures. However, these findings are preliminary and based on a single-institution cohort with a limited test set. Multi-centre validation, scanner-domain testing, failure-case analysis, uncertainty estimation, independent clinician review, and prospective workflow assessment are required before clinical translation can be firmly supported. Nevertheless, our results provide a solid foundation for future clinical validation of this cost-effective ART solution.

## Figures and Tables

**Table 1:** Quantitative comparison of automatic and manual delineation (Mean ± SD).

ROIs	Dice	HD95 (mm)	MSD (mm)
Bladder	0.95 ± 0.03	5.97 ± 4.15	1.24 ± 0.60
Rectum	0.83 ± 0.07	6.75 ± 4.08	1.91 ± 0.93
CTV	0.81 ± 0.04	8.34± 3.79	2.65 ± 0.98
CTV1	0.72 ± 0.14	6.92 ± 3.64	2.38 ± 1.40
Femurs	0.89 ± 0.15	6.54 ± 9.12	2.31 ± 3.58
Colon	0.48 ± 0.22	34.06 ± 36.49	11.43 ± 15.87
Intestine	0.24 ± 0.28	35.78 ± 23.02	17.35 ± 15.45

**Table 2:** Comparison of dosimetric parameters between adaptive plans and initial plans (mean ± SD or quartile).

ROIs	Parameter	Adaptive Plan	Initial Plan	95%CI	*p*
PTV	V_62Gy_ [%]	95.54 ± 0.49	88.97 ± 3.62	(5.12, 8.10)	<0.001
	HI	0.07 (0.05, 0.08)	0.24 (0.19, 0.38)	— *	<0.001
	CI	0.91 (0.89, 0.91)	0.71 (0.68, 0.75)	—	<0.001
PTV1	V_46Gy_ [%]	96.11 ± 0.88	91.38 ± 2.95	(2.15, 7.31)	0.008
	HI	0.22 ± 0.05	0.37 ± 0.10	(−0.24, −0.06)	0.003
	CI	0.60 ± 0.03	0.49 ± 0.05	(0.06, 0.16)	<0.001
Rectum	D_mean_ [cGy]	2250.33 ± 696.79	2638.70 ± 741.14	(−654.23, −122.51)	0.007
	V_50Gy_ [%]	9.36 ± 3.09	15.93 ± 9.33	(−10.84, −2.30)	0.003
	V_40Gy_ [%]	15.46 ± 5.04	22.70 ± 11.20	(−12.16, −2.32)	0.004
	V_30Gy_ [%]	21.17 (16.96, 35.58)	33.06 (23.78, 38.81)	—	0.030
Bladder	D_mean_ [cGy]	2170.21 ± 823.69	2383.66 ± 747.05	(−459.12, 32.22)	0.084
	V_50Gy_ [%]	12.76 ± 5.32	14.68 ± 6.35	(−4.42, 0.58)	0.120
	V_40Gy_ [%]	17.86 ± 6.64	21.40 ± 8.08	(−6.60, −0.48)	0.025
	V_30Gy_ [%]	26.68 ± 13.62	31.18 ± 12.27	(−9.09, 0.09)	0.058
Colon	D_mean_ [cGy]	277.30 (201.90, 3320.30)	312.80 (201.50, 3035.60)	—	0.629
Intestine	D_mean_ [cGy]	232.30 (82.40, 2271.85)	283.20 (84.35, 1896.60)	—	0.795

* For non-normally distributed variables, data are presented as median (IQR), and *p*-values are based on the Wilcoxon signed-rank test. 95% confidence intervals are not reported for these variables due to the non-parametric nature of the analysis.

**Table 3:** Comparison of the proposed integrated AI workflow with recently published adaptive radiotherapy frameworks.

Study	Platform	sCT Method	Segmentation Method	Adaptive Re-Planning	Integration Level	Key Feature
Eckl et al. [29]	C-arm linac	CycleGAN	Manual	✗	Partial	sCT generation only
Fu et al. [30]	C-arm linac	✗	nnU-Net	✗	Partial	Segmentation only
Dai et al.[32]	C-arm linac	CycleGAN	CNN	✗	Partial	sCT + segmentation (no re-planning)
Byrne et al. [33]	Ethos (dedicated)	Built-in	Built-in	✓	Full	Dedicated platform, high cost
Christiansen et al. [34]	MR-linac	MRI-based	Manual/AI	✓	Full	MR-dedicated platform
Preziosi et al. [35]	Ethos (dedicated)	Built-in	Built-in	✓	Full	Dedicated platform, high cost
Our study	C-arm linac	CycleGAN	nnU-Net	✓	Full	Cost-effective, no hardware upgrade

✓ indicates presence; ✗ indicates absence.

## Data Availability

Anonymized data are available from the corresponding author upon reasonable request from a qualified investigator.

## Appendix A

### Appendix A.1

Specifically, a “CycleGAN-ResNet” architecture was applied, employing a ResNet generator with nine residual blocks, along with a 70 × 70 PatchGAN discriminator. The model was trained using the Adam optimizer with β_1_ = 0.5 and β_2_ = 0.999. The initial learning rate was set to 2 × 10^−4^ with a linear decay schedule (multiplied by 0.9 every 10 epochs), and the batch size was 1. Training was conducted for 50 epochs. The loss function combined adversarial loss, cycle-consistency loss, and identity loss with a weighting of 1:10:5. No additional data augmentation was applied beyond the framework’s default. Model implementation was performed on an Ubuntu 18.04 LTS platform using an NVIDIA RTX 4090 GPU.

### Appendix A.2

In this study, nnU-Net was implemented using the PyTorch (version 2.5.1) framework on a Linux operating system via a Python application programming interface, with computational acceleration provided by an NVIDIA graphics processing unit. A global normalization scheme was applied based on image features, and the orientation of all images was unified to RAS. After preprocessing, the nnU-Net model was trained directly. Specific model parameters were automatically configured by the built-in trainer. The default architecture employed plain convolutions, instance normalization, and Leaky ReLU activation functions. Downsampling used strided convolutions, upsampling used transposed convolutions, and each resolution stage of the encoder and decoder had two convolutional blocks. nnU-Net also utilized five-fold cross-validation; the training dataset was automatically split into five folds to train five configurations, each using one subset as the validation dataset. After cross-validation, nnU-Net empirically selected the best-performing configuration or ensemble. After the population model was trained, when a new patient presented for the first time, the population model was lightly fine-tuned using the patient’s own pCT and physician-drawn manual contours as individual prior information. A smaller learning rate of 0.001 was used during fine-tuning, and only 15 epochs were trained to prevent overfitting. Other hyperparameters, such as the loss function, network architecture, and optimizer, remained consistent with those used for population model training.

## Appendix B

The quality of sCT images was evaluated against deformed CT images using peak signal-to-noise ratio (PSNR), mean absolute error (MAE), structural similarity index (SSIM) and gamma passing rate. The global 3D gamma passing rate was computed using Python 3.12.7 software with a low-dose threshold set at 10%. Auto-segmentation accuracy was evaluated by comparing automated contours with manually refined contours, using Dice Similarity Coefficient (DSC), 95% Hausdorff Distance (HD95), and Mean Surface Distance (MSD). Detailed calculation methods for these metrics are provided below:

$$PSNR=10\times {log}_{10}\left(\frac{{MAX}^{2}}{MSE}\right)$$

where MAX represents the maximum possible signal intensity, and MSE is the mean squared error of the image.

$$MAE=\frac{1}{n_{x}n_{y}n_{z}}\sum_{I,j,k}^{n_{x}n_{y}n_{z}}\left|S\left(i,j,k\right)-D\left(i,j,k\right)\right|$$

where $S\left(i,j,k\right)$ and $D\left(i,j,k\right)$ are the values of the pixels $\left(i,j,k\right)$ in the sCT and deformed CT images, respectively, and $n_{x}$, $n_{y}$ and $n_{z}$ are the number of voxels in the x, y, and z directions of the image.

$$SSIM=\frac{1}{N}\sum_{k=1}^{N}\frac{\left(\left.2\mu_{S_{k}}\mu_{D_{k}}+C_{1}\right)\right.\left.\left(2\sigma_{S_{k}D_{k}}+C_{2}\right.\right)}{\left(\left.\mu_{S_{k}}^{2}+\mu_{D_{k}}^{2}+C_{1}\right)\right.\left.\left(\sigma_{S_{k}}^{2}\right.+\sigma_{D_{k}}^{2}+C_{2}\right)}$$

where $\mu_{S_{k}}$ and $\mu_{D_{k}}$ are the mean values of the sCT and deformed CT images at slice k, respectively. $\sigma_{S_{k}}$ and $\sigma_{D_{k}}$ are the standard deviations, and $\sigma_{S_{k}D_{k}}$ is the covariance. $C_{1}={\left(0.01L\right)}^{2}$ and $C_{2}={\left(0.03L\right)}^{2}$, and L is set to 1. $C_{1}$ and $C_{2}$ are constants added to stabilize the calculation and prevent division by zero. N is the number of effective slices participating in the calculation.

$$\mathrm{DSC}=\frac{2\left|X\cap Y\right|}{\left|X\right|+\left|Y\right|}\;$$

where, $\left|X\right|$ and $\left|Y\right|$ are the cardinalities of the ground truth mask X and predicted mask Y, respectively.

$$\mathrm{HD}95=\max\left[{\vec{d}}_{H,95}\left(X,Y\right),{\vec{d}}_{H,95}\left(Y,X\right)\right]$$

where ${\vec{d}}_{H,95}\left(X,Y\right)$ is 95% percentile Hausdorff distance that measures the 95th percentile distance of all distances between points in X and the nearest point in Y, defined as ${\vec{d}}_{H,95}\left(X,Y\right)=K_{95}\left(\min_{y\in \left|Y\right|}d\left(x,y\right)\right)$, and $d\left(x,y\right)$ is Euclidean distance.

$$\mathrm{MSD}=\frac{1}{\left|X\right|+\left|Y\right|}\left(\sum_{x\epsilon X}\vec{d}\left(x,Y\right)+\sum_{y\epsilon Y}\vec{d}\left(y,X\right)\right)$$

where $\vec{d}\left(x,Y\right)=\min_{y\in \left|Y\right|}d\left(x,y\right)$.
